# Bacteremic Typhoid Fever in Children in an Urban Slum, Bangladesh

**DOI:** 10.3201/eid1102.040422

**Published:** 2005-02

**Authors:** W. Abdullah Brooks, Anowar Hossain, Doli Goswami, Amina Tahia Sharmeen, Kamrun Nahar, Khorshed Alam, Noor Ahmed, Aliya Naheed, G. Balakrish Nair, Stephen Luby, Robert F. Breiman

**Affiliations:** * ICDDR,B Centre for Health and Population Research, Dhaka, Bangladesh

**Keywords:** *Salmonella* Typhi, typhoid fever, preschool children, population-based, surveillance, incidence, relative risk, antimicrobial, resistance, Bangladesh, dispatch

## Abstract

We confirmed a bacteremic typhoid fever incidence of 3.9 episodes/1,000 person-years during fever surveillance in a Dhaka urban slum. The relative risk for preschool children compared with older persons was 8.9. Our regression model showed that these children were clinically ill, which suggests a role for preschool immunization.

Typhoid fever is a major cause of illness; the global incidence in 2000 was an estimated 21,650,974 cases with 216,510 deaths (*1*). The cause of typhoid fever, *Salmonella enterica* subspecies *enterica* serotype Typhi (*S*. Typhi), is both waterborne and foodborne, with an annual incidence approaching 1% in disease-endemic areas (*2**–**4*). Peak incidence is reported to occur in children 5–15 years of age; however, in regions where the disease is highly endemic, children <5 years of age may have among the highest infection rates (*1**,**4**–**6*). Population-based data are limited (*1*) and would be helpful for refining estimates of the impact of disease and for identifying age groups at highest risk, thereby making it possible to optimize vaccination strategies (*7**,**8*).

Data on disease severity and sequelae can contribute to estimating the impact of disease. Most complications—including intestinal perforation and peritonitis, encephalopathy, intestinal hemorrhage, hepatosplenomegaly, vomiting, and diarrhea (*4**,**9*)—are late onset. Whether children <5 years of age (preschool children) have silent infection or clinical disease is controversial (*4**,**5**,**10*), which has important implications for both case management and prevention. We report our findings from prospective, population-based active surveillance.

## The Study

Since 1998, the ICDDR,B Centre for Health and Population Research has operated a surveillance and intervention site in Kamalapur, an urban slum in Dhaka, Bangladesh. We initiated fever surveillance for dengue fever and dengue hemorrhagic fever in August 2000. To identify treatable causes of fever, we obtained blood cultures from December 6, 2000, to October 8, 2001.

The community comprises 7 geographic strata, representing 379 clusters. We selected the surveillance cohort by using stratified cluster randomization and obtained informed written consent from all households.

Field research assistants screened household members for fever in their homes once weekly with a standardized questionnaire. We defined fever as >3 consecutive febrile days (reported) for persons >5 years of age, or any duration of fever for preschool children (<5 years of age). This definition facilitated detection of dengue fever. Field research assistants referred febrile participants to our field clinic, where study physicians confirmed fever and collected clinical data by using a standard form. Patients with an axillary temperature of >38°C were designated as febrile. After collecting blood for serologic tests of dengue and dengue hemorrhagic fever, we collected an additional 1 mL of blood from preschool children and >3 mL from older persons for culture.

Blood cultures were transported within 2 hours to our clinical microbiology laboratory (12 km from the field clinic). Specimens were processed by using standard methods with in-tube lysis centrifugation (Wampole isolator 1.5, Carter-Wallace, Inc., Cranbury, NJ, USA), plated on blood, chocolate, and MacConkey agar and incubated at 37°C for 16 to 18 hours. Colonies were evaluated with biochemical tests and confirmed by serologic identification with commercial antisera (Denka, Sieken, Co., Ltd., Tokyo, Japan). Antimicrobial susceptibility was determined by disk diffusion using standard NCCLS methods (*11*).

We confirmed typhoid fever if we isolated *S*. Typhi from blood during a febrile episode. Febrile controls were culture-negative for *S*. Typhi, Paratyphi, or *Salmonella* group D during fever.

If *S*. Typhi was isolated, then we treated the infection with 14 days of standard therapy, adjusting for antimicrobial susceptibility. First-line drugs were amoxicillin (40 mg/kg up to 1,500 mg orally divided 3 times daily) or cotrimoxazole (10 mg/kg trimethoprim divided into 2 daily doses). When patients remained febrile after 72 hours or new danger signs (e.g., lethargy, inability to drink, cyanosis, convulsions), developed, treatment was considered to have failed. We treated treatment failure in persons >12 years of age with ciprofloxacin (500 mg orally twice a day) and referred younger patients to the hospital. We defined recovery as >7 consecutive afebrile days after completing therapy.

Statistical analysis was performed by using Stata/SE Release 8.2 (Stata Statistical Software: Release 8.0. 2003, Stata Corporation, College Station, TX, USA). Incidence was determined by dividing the number of cases by person-years of observation, with calculation of exact 95% confidence intervals (CIs). Univariate analysis was performed by using 2-by-2 tables for relative odds (RO) and 95% CIs. We obtained p values by using the Fisher 2-tailed exact test. Multivariate modeling was conducted by stepwise forward logistic regression, using all covariates significantly associated with typhoid fever in univariate analysis. Covariates that were significant when age, sex, and geographic location were controlled for, were retained in the final model. We adjusted models for clustering of repeat patient visits and tested for goodness-of-fit with either Pearson or Hosmer-Lemeshow methods (*12*). Research Review and Ethical Review Committees of ICDDR,B approved this study.

During the study period, we took blood for culture from 888 (99.9%) of 889 eligible study participants; 54 (6.1%) reported prior medication exposure. All specimens had adequate volume. A microorganism was isolated from 65 (7.3%) cultures. Isolation rates were highest in winter. No positive culture reported >1 organism (Table 1), nor did any culture-positive patient have laboratory-confirmed dengue.

**Table 1:** Distribution of 65 blood culture isolates

Organism	No. (%)	Cumulative (%)
*Salmonella* Typhi	49 (75.4)	75.4
*Staphylococcus epidermidis*	2 (3.1)	78.5
*Acinetobacter* spp.	4 (6.2)	84.6
*Salmonella* group D	2 (3.1)	87.7
Viridans-group *Streptococcus*	2 (3.1)	90.8
*Salmonella* Paratyphi A	3 (4.6)	95.4
*Streptococcus pneumoniae*	2 (3.1)	98.5
*Enterobacter* spp.	1 (1.5)	100.0

**Figure 1:**
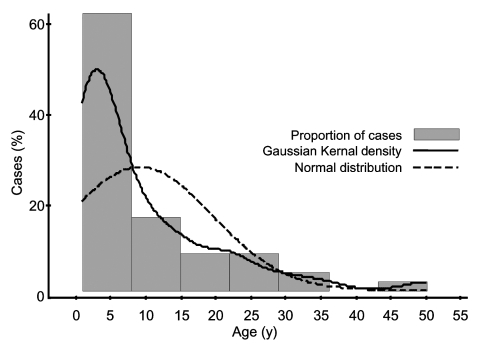
Distribution of typhoid fever by age.

**Figure 2:**
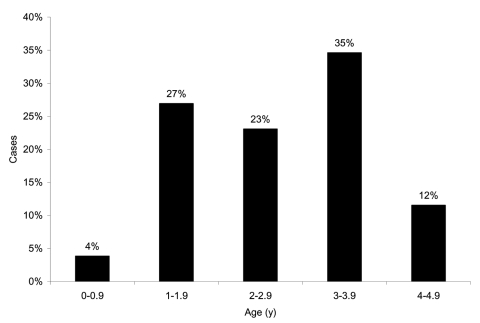
Age distribution of patients <5 years of age with typhoid fever.

*S*. Typhi was isolated from 26 preschool children (Figure 1) and 23 older study participants (age range 10 months–50 years, median 4.0 years [95% CI 3.0–8.0]). There were 1,393 person-years of observation for preschool children and 11,014 for others. Overall, typhoid fever incidence was 3.9 episodes/1,000 person-years. Typhoid fever incidence among preschool children was 18.7 episodes/1,000 person-years and 2.1 episodes/1,000 person-years among older participants. The incidence rate difference between the 2 age groups was 16.6 cases/1,000 person-years (95% CI 9.4–23.8; p < 0.001). Preschool children's relative risk for typhoid fever was thus 8.9 (95% CI 4.9–16.4). Typhoid fever among preschool children varied by age, with 4% in the first year of life and 85% occurring in those 2 to 4 years of age (Figure 2).

We investigated surveillance bias resulting from fever definition differences between age groups (*4*). Preschool children's mean fever duration (days) prior to visiting the clinic was 4.0 (95% CI 3.2–4.8) and other patients' mean duration was 4.9 (95% CI 2.9–6.8, p = 0.37). We collected 84.6% of preschool specimens and 78.3% of others' after 3 febrile days, and 96.2% and 86.7%, respectively, by day 7.

A multivariate model showed that typhoid fever patients were more likely than febrile controls to be preschool age (RO 2.04; 95% CI 1.09–3.82; p = 0.03), have >3 days of fever (RO 2.55; 95% CI 1.16–5.63; p = 0.02), have temperature >39°C (RO 1.95; CI 1.01–3.80; p = 0.04), and have mental status changes (RO 3.94; CI 1.98–7.81; p < 0.02). Another model indicated preschool typhoid fever patients were significantly more likely than older patients to have fever >39°C (RO 1.62; CI 1.21–2.17), mental status changes (RO 3.54; CI 2.25–5.55), and crepitations (rales) on auscultation (RO 4.44; CI 3.11–6.33).

All patients with culture-confirmed typhoid fever recovered, except for 1 child with tuberculosis. Four adults required ciprofloxacin. No hospitalizations, complications, or deaths occurred among confirmed typhoid fever patients.

In vitro antimicrobial susceptibility testing (Table 2) showed a high prevalence of ampicillin, cotrimoxazole, and chloramphenicol resistance, with 27 isolates (55.1%) resistant to all 3; ceftriaxone resistance was found in isolates from 1 preschool child. Routine nalidixic acid testing was not performed, following NCCLS 2000 guidelines.

**Table 2:** Antimicrobial resistance patterns of *Salmonella enterica* serovar Typhi, Kamalapur, 2001

Antimicrobial agent	% resistance
Ampicillin	55.1
Cotrimoxazole	57.1
Chloramphenicol	57.1
Ciprofloxacin	0.0
Ceftriaxone	2.0

## Conclusions

Our data indicate a high infection ratio in this urban population, which is highest among preschool children. These ratios are comparable to recent regional reports (*4**,**6**,**13*) and indicate that typhoid fever in preschool children may be underappreciated. That preschool children have 8.9 times the risk for *S*. Typhi infection as older persons corroborates age-specific rates in highly disease-endemic areas (*1*). The antimicrobial susceptibility data indicate high ratios of in vitro resistance to standard antimicrobial agents, with a high prevalence of multidrug resistance.

The degree of illness of preschool children is controversial; some report benign bacteremia (*5**,**14*) and others have found clinical illness (*4**,**13*). Our multivariate model shows that preschool children are clinically ill. Coexisting conditions, particularly pneumonia, are not only more common in preschool typhoid fever patients but also may result in misclassification and underreporting, as well as contribute to a worsening cycle of repeated infection and deaths. Future studies should explore these issues in this age group.

Substantial clinical illness among preschool children argues the need for them to be enrolled in vaccination programs. The age-specific infection rates suggest vaccination in the first year of life, integrating with existing Expanded Programme on Immunization (EPI) schedules. This practice would require either a polysaccharide protein-conjugate vaccine to stimulate T-cell–dependent responses (*15*) or a live attenuated oral vaccine, since T-cell–independent responses do not mature until the child is 18–24 months of age.

The limitations of this study could result in an underestimate of the incidence of typhoid fever. First, this study was not designed to measure typhoid fever incidence or disease impact. The surveillance program was designed to identify dengue. Thus, febrile episodes for young children were defined differently than for older persons. Although we did not find evidence of preferential selection for young children, future studies may adopt a common fever definition. Second, the blood volume examined, though not inadequate, may not have been optimal. Third, blood culture sensitivity is relatively low, estimated at 25%–50% (*1*). Fourth, the 6.1% estimate of earlier medicine exposure may be an underestimate, as we did not validate these reports. If these agents were antimicrobial, the number of serovar Typhi isolates recovered from peripheral blood would be reduced. Fifth, we had only 10 months of observation and therefore did not attempt an estimate of disease impact, adjustments for blood culture sensitivity, or exposure to antimicrobial agents. Ours is thus a conservative estimate of incidence. Further observation should allow the impact of disease to be estimated.
